# Long‐term (beyond 5 years) clinical impact of Xience everolimus‐eluting stent implantation

**DOI:** 10.1002/hsr2.365

**Published:** 2021-09-08

**Authors:** Hiroaki Matsuda, Yoriyasu Suzuki

**Affiliations:** ^1^ Department of Cardiology Nagoya Heart Center Nagoya Japan

## Abstract

**Objects:**

We aim at examining the long‐term clinical outcome after Xience everolimus‐eluting stent (X‐EES) implantation.

**Background:**

Long‐term clinical outcomes beyond 5 years after X‐EES implantation remain unclear.

**Methods:**

This retrospective study has collected data from 1184 consecutive patients, corresponding to 1463 lesions, who were treated with X‐EES alone in the Nagoya Heart Center between January 2010 and December 2013. The primary endpoint was the 10‐year cumulative incidence of target lesion failure (TLF), defined as cardiac death, target vessel myocardial infarction (MI), and clinically indicated target lesion revascularization (TLR). Definite/probable stent thrombosis (ST) was evaluated as a secondary outcome.

**Results:**

At 10 years, the cumulative incidence of TLF was recorded to be 12.4%, whereas that of cardiac death, target vessel MI, and clinically indicated TLR was at 4.4%, 4.1%, and 7.8%, respectively. The cumulative rate of definite/probable ST was observed to remain low (0.3% at 30 days; 0.3% at 1 year; 0.6% at 5 years; and 1.1% at 10 years). In the multivariate analysis, the risk factors of TLF were insulin‐treated diabetes (hazard ratio (HR), 1.93; 95% confidence interval (CI), 1.13‐3.29; *P* = .02), left ventricular dysfunction (HR, 2.28; 95% CI, 1.43‐3.62; *P* < .01), hemodialysis (HR, 2.22; 95% CI, 1.39‐3.56; *P* < .01), prior percutaneous coronary intervention (HR, 1.68; 95% CI, 1.18‐2.41; *P* < .01), peripheral vascular disease (HR, 1.70; 95% CI, 1.07‐2.69; *P* < .01), severe calcification (HR, 2.08; 95% CI, 1.36‐3.09; *P* < .01), and in‐stent restenosis (HR, 2.93; 95% CI, 1.64‐4.89; *P* < .01).

**Conclusions:**

The incidence rates of the long‐term adverse effects after X‐EES implantation, such as late TLR and ST, were determined to be low in this study; however, they increased over time until 10 years after stent implantation.

## 1 INTRODUCTION

Drug‐eluting stents (DESs) have been seen to reduce the risk of restenosis compared with bare‐metal stents (BMS).^1^, ^2^, ^3^ However, real‐world clinical studies on first‐generation DES implants have revealed some of their long‐term adverse effects such as very late stent thrombosis (VLST) and late target lesion revascularization (TLR).^4^, ^5^ The Xience everolimus‐eluting stent (X‐EES) has been the most widely used second‐generation DES in Japan; compared with the first‐generation DES, X‐ESS is associated with superior safety and efficacy outcomes.^6^, ^7^, ^8^ Also, compared with the other second‐generation DES, the X‐ESS has never been reported to be superiority to safety and efficacy in mid‐ to long term.^9^, ^10^ However, the occurrence rate of its long‐term (beyond 5 years) clinical effects, including its risk factors, remains unclear. Therefore, this retrospective cohort study investigated the long‐term safety and efficacy of X‐EES.

## 2 METHODS

### 2.1 Patient population

Between January 2010 and December 2013, 1184 consecutive patients with 1463 lesions have undergone percutaneous coronary intervention (PCI) with alone at the Nagoya Heart Center. All patients gave their written informed consent for the procedure and the follow‐up protocol, and the study has also received approval from the ethics committee of our hospital.

### 2.2 Procedures

The PCI strategy was left to the discretion of the operating surgeon. Patients who were scheduled for PCI received daily oral aspirin (≥81 mg/day) and P2Y12 inhibitor (75 mg/day clopidogrel), while ticlopidine (200 mg/day) was administered only in those who cannot tolerate clopidogrel. Patients with acute coronary syndromes, namely, STEMI (ST elevation myocardial infarction) and NSTE‐ACS (non‐ST elevation acute coronary syndrome), received loading doses of aspirin (200 mg) and P2Y12 inhibitor (300 mg clopidogrel). During the procedure, unfractionated heparin (100 U/kg) was administered to all the patients who reached an activated clotting time of 250 seconds. After the procedure, all patients were prescribed with optimal pharmacologic therapy based on the current guidelines, including statins, beta‐blockers, or renin‐angiotensin system blockers. Moreover, the duration of dual antiplatelet therapy (DAPT) was also dependent on the surgeon's discretion.

### 2.3 Data collection and clinical follow‐up

All the patients were followed up at 1, 3, 6, and 12 months after their index procedure and annually thereafter. Additional information was obtained by telephone communication or medical records, as needed, and follow‐up angiography was recommended in patients based on clinical symptoms and findings of noninvasive tests.

### 2.4 Endpoints and definitions

The primary endpoint of this study was the target lesion failure (TLF) up to 10 years after the index procedure, which is defined as a composite of cardiac death, target vessel myocardial infarction (MI), and clinically indicated TLR. The secondary endpoint included individual components of the composite primary endpoints and definite/probable stent thrombosis (ST) at various time points. Risk factors of clinically indicated TLR and definite/probable ST rate in the cases of acute MI (AMI) were also evaluated.

Death was, by default, considered as cardiac unless an unequivocal noncardiac cause could be established. MI and ST were defined as per the Academic Research Consortium.^11^ TLR was defined as either PCI or coronary artery bypass grafting due to restenosis or thrombosis of the target lesion that included both proximal and distal edge segments (within 5 mm) as well as the ostium of the side branches. Clinically driven TLR was defined as TLR performed because of ischemic symptoms, electrocardiographic changes at rest, or positive stress test results.

### 2.5 Statistical analyses

Categorical variables are presented as numbers and percentages, whereas the continuous variables are expressed as mean ± SD or median with inter‐quartile range. Cumulative incidence was estimated using the Kaplan‐Meier method, and differences were assessed. For TLF and clinically indicated TLR, we used landmark analysis with 1‐year intervals to examine the late events after the first year. Patients with individual endpoint events before 1 year were excluded in the landmark analysis. A Cox proportional hazards model was used to identify the independent risk factors for clinically indicated TLR during the follow‐up period of 10 years. Continuous variables were based on clinically meaningful reference values. To determine the independent risk factors, variables that were significant (*P* < .10) after univariate analysis were subjected to multivariable modeling, and adjusted hazard ratios and their 95% confidence intervals were then calculated; a *P*‐value of <.05 was considered significant. Statistical analyses were performed using JMP version 14.0 (SAS Institute Inc., Cary, North Carolina, USA).

## 3 RESULTS

### 3.1 Baseline characteristics

Baseline patient, lesion, and procedural characteristics are summarized in Tables 1 and 2. High‐risk factors such as diabetes mellitus, hemodialysis, prior MI, prior PCI, and peripheral vascular diseases were also found prevalent in the study population (Table 1), as were complex lesion characteristics (AHA/ACC type B2/C lesions) such as bifurcation, diffuse lesion, severe calcification, chronic total occlusion, and in‐stent restenosis (Table 2). The median follow‐up duration was 3277 days (first and third quartiles [Q1‐Q3]: 2574‐3677), and the 10‐year clinical follow‐up was completed in 1133 (95.7%) of the 1184 patients.

**TABLE 1:** Patient characteristics according to the occurrence of TLF

	Overall	TLF (+)	TLF (−)	*P* Value
	N = 1184	N = 127	N = 1057	
Median follow‐up duration (days)	3277	2808	3313	<.01
Age (years)	68.3 ± 10.1	69.5 ± 9.2	68.2 ± 10.2	.2
≧75 years	364 (30.7%)	37 (29.1%)	327 (30.9%)	.7
Female gender	305 (25.8%)	32 (25.2%)	234 (25.8%)	.4
Body mass index	23.9 ± 3.4	23.7 ± 3.4	24.0 ± 3.3	.3
Hypertension	928 (78.4%)	106 (83.5%)	822 (77.8%)	.1
Dyslipidemia	870 (73.5%)	97 (76.4%)	773 (73.1%)	.4
Diabetes mellitus	508 (42.9%)	62 (48.8%)	446 (42.2%)	.2
Insulin‐treated diabetes	66 (5.6%)	16 (12.6%)	50 (4.7%)	<.01
Treated with oral medication only	353 (29.8%)	38 (29.9%)	315 (29.8%)	1.0
Treated with diet therapy only	90 (7.6%)	8 (6.3%)	82 (7.8%)	.6
Current smoker	272 (23.0%)	29 (22.8%)	243 (23.0%)	1.0
Creatinine (mg/dL)	1.51 ± 2.07	1.83 ± 2.23	1.21 ± 1.47	<.01
Hemodialysis	95 (8.0%)	24 (18.9%)	71 (6.7%)	<.01
Ejection fraction (%)	57.3 ± 14.5	53.9 ± 15.9	57.7 ± 14.3	<.01
Left ventricular dysfunction (LVEF <35%)	106 (9.0%)	22 (17.3%)	84 (7.9%)	<.01
Clinical presentation				
Stable coronary artery disease	1006 (85.0%)	107 (84.3%)	899 (83.4%)	.8
Unstable angina	48 (4.0%)	9 (7.1%)	39 (3.9%)	.09
Acute myocardial infarction	130 (11.0%)	11 (8.7%)	119 (12.6%)	.2
Prior myocardial infarction	283 (23.9%)	38 (29.9%)	245 (23.2%)	.09
Prior percutaneous coronary intervention	506 (42.7%)	72 (56.7%)	434 (41.1%)	<.01
Prior coronary‐artery bypass grafting	40 (3.4%)	7 (5.5%)	33 (3.1%)	.2
Peripheral vascular disease	139 (11.7%)	25 (19.7%)	114 (10.8%)	<.01
Prior stroke	143 (12.1%)	16 (12.6%)	127 (12.1%)	.9
Atrial fibrillation	99 (8.4%)	12 (9.4%)	87 (8.2%)	.2
Medications				
Dual antiplatelet therapy	1176 (99.3%)	126 (99.2%)	1050 (99.4%)	.8
Aspirin	1177 (99.4%)	126 (99.2%)	1051 (99.5%)	.6
Thienopyridines	1174 (99.2%)	127 (100.0%)	1049 (99.2%)	.2
Clopidogrel	1120 (94.7%)	115 (90.6%)	1005 (95.2%)	.03
Ticlopidine	56 (4.7%)	12 (9.4%)	44 (4.2%)	<.01
Anticoagulants	106 (9.0%)	8 (10.3%)	98 (9.9%)	.5
Warfarin	103 (8.7%)	15 (11.8%)	88 (8.5%)	.2
Direct‐acting oral anticoagulants	1 (0.8%)	0 (0.0%)	1 (0.9%)	.7
B‐blockers	333 (28.1%)	33 (26.0%)	300 (28.4%)	.6
ACE‐I/ARB	726 (61.3%)	82 (64.6%)	644 (58.1%)	.2
Calcium‐channel blockers	462 (39.1%)	53 (41.7%)	409 (38.7%)	.5
Statins	888 (75.1%)	85 (66.9%)	803 (76.0%)	.02

*Note*: Values are expressed as mean ± SD or number (%).

Abbreviations: ACE‐I, angiotensin converting enzyme inhibitors; ARB, angiotensin II receptor blockers; LVEF, left ventricular ejection fraction; TLF, target lesion failure.

**TABLE 2:** Lesion and procedural characteristics according to the occurrence of TLF

	Overall	TLF (+)	TLF (−)	
	N = 1463	N = 144	N = 1319	*P* Value
Target‐vessel location				
Left main coronary artery	59 (4.0%)	7 (4.9%)	52 (3.9%)	.6
Left anterior descending coronary artery	669 (45.8%)	60 (41.7%)	609 (46.2%)	.3
Left circumflex coronary artery	294 (20.1%)	27 (18.8%)	267 (20.2%)	.7
Right coronary artery	439 (30.0%)	49 (34.0%)	390 (30.0%)	.3
Bypass graft	2 (0.1%)	1 (0.7%)	1 (0.1%)	.06
Number of treated lesions per patient	2.04 ± 0.78	2.16 ± 0.78	2.03 ± 0.78	.04
Ostium	87 (5.9%)	11 (7.6%)	76 (5.8%)	.4
Bifurcation	499 (34.1%)	48 (33.3%)	451 (34.2%)	.8
Diffuse lesion (lesion length > 20 mm)	473 (32.3%)	48 (33.3%)	425 (32.2%)	.7
Severe tortuosity	95 (6.5%)	10 (6.9%)	85 (6.4%)	.8
Severe calcification	193 (13.2%)	30 (20.8%)	163 (12.4%)	<.01
Thrombus	69 (5.4%)	15 (10.4%)	54 (9.5%)	.7
Ulceration	46 (3.3%)	4 (2.8%)	42 (5.0%)	.2
Chronic total occlusion	148 (10.1%)	14 (9.7%)	134 (10.2%)	.9
In‐stent restenosis	68 (4.6%)	15 (10.4%)	53 (4.0%)	<.01
ACC/AHA lesion type B2/C	1090 (74.7%)	115 (79.9%)	975 (74.1%)	.1
Number of stents used per patient	1.22 ± 0.51	1.22 ± 0.45	1.21 ± 0.51	.9
Stent diameter (mm)	2.89 ± 0.35	2.90 ± 0.37	2.88 ± 0.35	.7
Total stent length per patient (mm)	26.2 ± 14.1	25.3 ± 12.1	26.3 ± 14.3	.4
Post dilatation	1035 (70.7%)	105 (72.9%)	930 (70.5%)	.5
Balloon size (mm)	3.04 ± 0.49	2.98 ± 0.43	3.04 ± 0.50	.2
Maximal inflation pressure (atm)	19.0 ± 4.6	18.7 ± 5.1	19.1 ± 4.6	.4
Distal protection	161 (11.0%)	18 (12.5%)	143 (10.8%)	.5
Thrombectomy	115 (7.9%)	12 (8.3%)	103 (7.8%)	.8
Imaging device used	1463 (100.0%)	144 (100.0%)	1319 (100.0%)	1.0
Rotablator used	181 (12.4%)	25 (17.4%)	156 (11.8%)	.06

*Note*: Values are expressed as mean ± SD or number (%).

Abbreviations: ACC/AHA, American College of Cardiology/American Heart Association; TLF, target lesion failure.

### 3.2 Clinical outcomes

The cumulative incidence of each clinical event for the 10‐year period is summarized in Table 3.

**TABLE 3:** Clinical outcomes up to 10 years

	No. of patients with at least one event		
	(Cumulative incidence)		
	1 year	5 years	10 years
All‐cause death	31 (2.8%)	108 (9.8%)	189 (18.2%)
Cardiac death	16 (1.4%)	31 (2.8%)	45 (4.4%)
Noncardiac death	15 (1.3%)	77 (7.2%)	144 (14.4%)
Clinically indicated target lesion revascularization	20 (1.8%)	59 (5.5%)	78 (7.8%)
Clinically indicated target vessel revascularization	29 (2.6%)	88 (8.2%)	122 (12.2%)
Any myocardial infarction	10 (0.9%)	35 (3.2%)	59 (6.0%)
Target vessel myocardial infarction	7 (0.6%)	26 (2.4%)	41 (4.1%)
Stent thrombosis	7 (0.6%)	19 (1.9%)	33 (3.3%)
Definite/Probable stent thrombosis	4 (0.3%)	7 (0.6%)	11 (1.1%)
Definite stent thrombosis	2 (0.2%)	5 (0.5%)	9 (1.0%)

*Note*: Values are expressed as number (%). Cumulative incidences of events are calculated by the Kaplan–Meier method.

**FIGURE 1:**
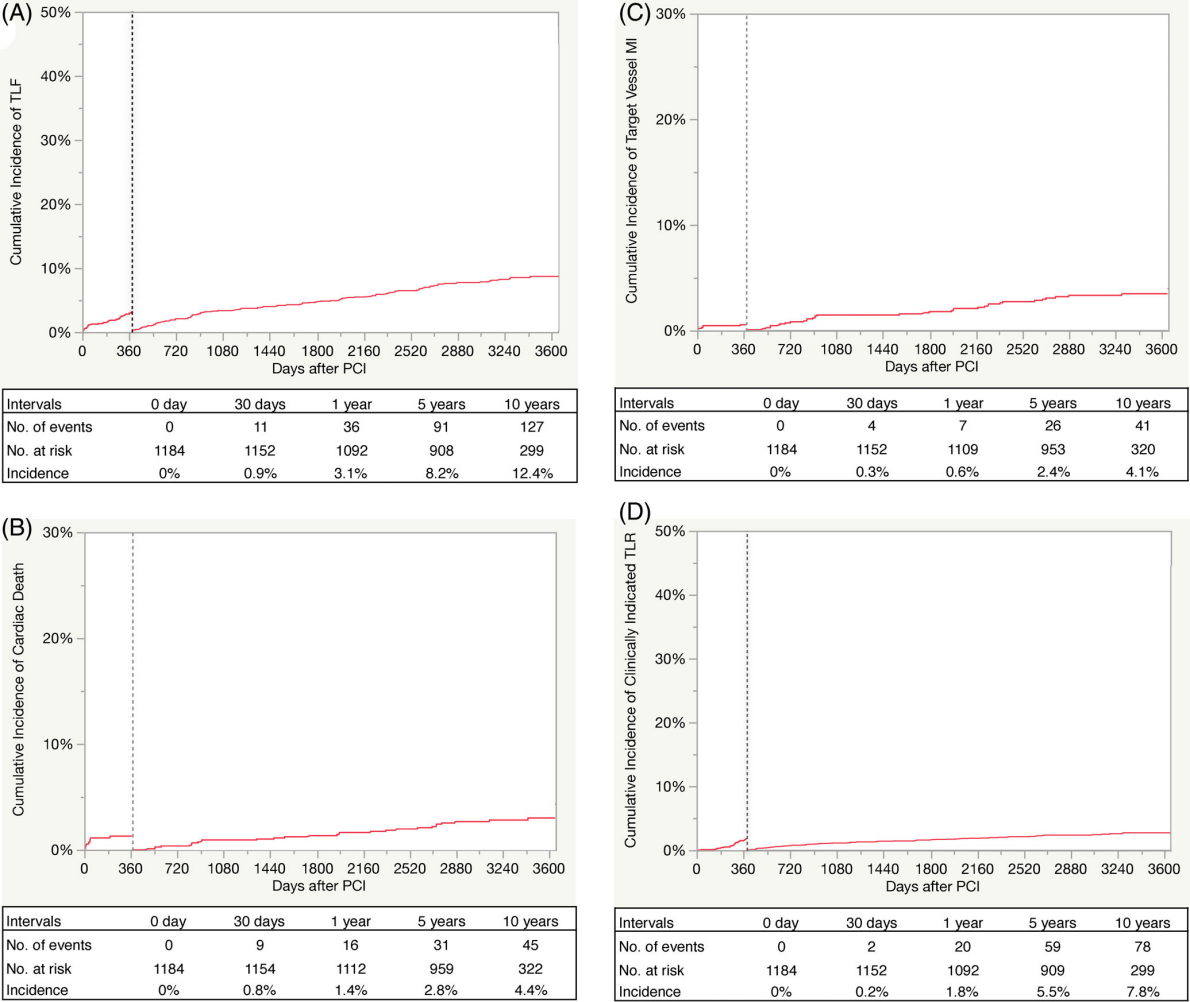
Cumulative incidence of the primary endpoint and its individual components up to 10 years. A, TLF; B, cardiac death; C, target vessel MI; D, clinically indicated TLR

TLF after X‐EES implantation was observed to occur in 36 patients (3.1%) at 1 year, in 91 patients (8.2%) at 5 years, and in 127 patients (12.4%) at 10 years (Table 3, Figure 1A). The cumulative incidence of each individual component of TLF at 10 years was determined to be at 4.4%, 4.1%, and 7.8% for cardiac death, target vessel MI, and clinically indicated TLR, respectively (Table 3, Figure 1B‐D). As shown in the landmark analysis, both the TLF and individual components of TLF were found to continue increasing gradually after the first year.

**FIGURE 2:**
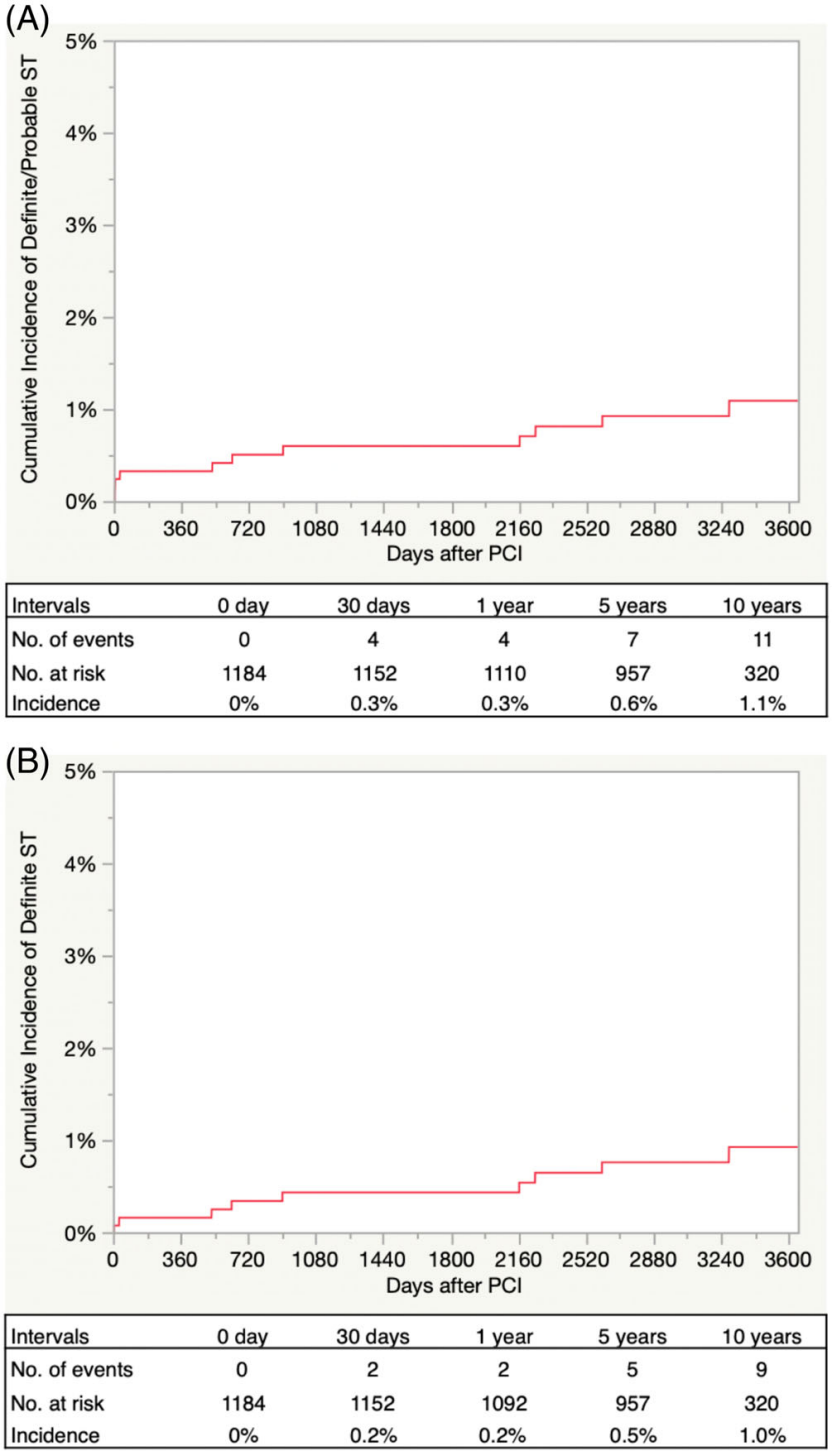
Cumulative incidence of the secondary up to 10 years. A, Definite/probable ST; B, definite ST

**FIGURE 3:**
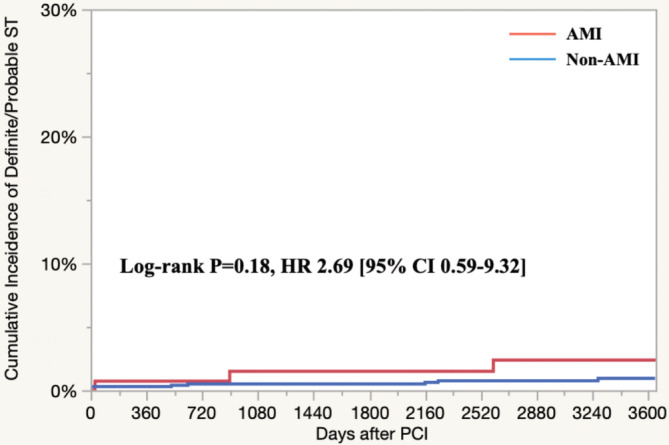
Subanalysis of the definite/probable ST: AMI vs non‐AMI

For secondary endpoints, the cumulative 10‐year incidence of definite/probable ST was determined to be at 0.3% at 1 year, 0.6% at 5 years, and 1.1% at 10 years (Table 3, Figure 2). There was no significant difference in the definite/probable ST rates among AMI and non‐AMI cases (Figure 3).

### 3.3 Risk factors of TLF

We used multivariate analysis to identify the risk factors for TLF. Insulin‐treated diabetes (hazard ratio (HR), 1.93; 95% confidence interval (CI), 1.13‐3.29; *P* = .02), left ventricular dysfunction (HR, 2.28; 95% CI, 1.43‐3.62; *P* < .01), hemodialysis (HR, 2.22; 95% CI, 1.39‐3.56; *P* < .01), prior percutaneous coronary intervention (HR, 1.68; 95% CI, 1.18‐2.41; *P* < .01), peripheral vascular disease (HR, 1.70; 95% CI, 1.07‐2.69; *P* < .01), severe calcification (HR, 2.08; 95% CI, 1.36‐3.09; *P* < .01), and in‐stent restenosis (HR, 2.93; 95% CI, 1.64‐4.89; *P* < .01) were considered significant throughout the follow‐up (Table 4).

**TABLE 4:** Univariate and multivariate cox models for TLF

	Univariate		Multivariate	
	HR (95% CI)	*P* value	HR (95% CI)	*P* value
Diabetes mellitus treated with insulin	2.63 (1.50‐4.31)	<.01	1.93 (1.13–3.29)	.02
Left ventricular dysfunction (LVEF <35%)	2.66 (1.64‐4.14)	<.01	2.28 (1.43–3.62)	<.01
Hemodialysis	3.46 (2.17‐5.30)	<.01	2.22 (1.39‐3.56)	<.01
Prior percutaneous coronary intervention	1.76 (1.24‐2.51)	<.01	1.68 (1.18–2.41)	<.01
Peripheral vascular disease	2.17 (1.37‐3.31)	<.01	1.70 (1.07–2.69)	<.01
Ticlopidine use instead of clopidogrel	2.29 (1.20‐3.98)	.01	1.73 (0.94‐3.15)	.08
Statins nonuse	1.63 (1.12‐2.35)	.01	1.47 (1.00‐2.16)	.05
Severe calcification	2.23 (1.47‐3.30)	<.01	2.08 (1.36–3.09)	<.01
In‐stent restenosis	2.48 (1.39‐4.10)	<.01	2.93 (1.64–4.89)	<.01

Abbreviations: CI, confidence interval; HR, hazard ratio; LVEF, left ventricular ejection fraction; TLF, target lesion failure.

### 3.4 Risk factors of clinically indicated TLR

We used multivariate analysis to identify the risk factors for clinically indicated TLR and insulin‐treated diabetes (HR, 2.03; 95% CI, 1.01‐3.69; *P* = .04), hemodialysis (HR, 2.48; 95% CI, 1.38‐4.27; *P* < .01), peripheral vascular disease (HR, 2.28; 95% CI, 1.35‐3.73; *P* < .01), and in‐stent restenosis (HR, 3.17; 95% CI, 1.58‐5.23; *P* < .01) as they are considered significant throughout the follow‐up (Table 5). In this cohort, lesion and procedural characteristics, except in‐stent restenosis, were not independent risk factors of clinically indicated TLR (Table 1).

**TABLE 5:** Univariate and multivariate cox models for CI‐TLR

	Univariate		Multivariate	
	HR (95% CI)	*P* value	HR (95% CI)	*P* value
Hypertension	1.86 (1.03‐3.71)	.04	1.41 (0.77‐2.85)	.28
Diabetes mellitus treated with Insulin	2.61 (1.31‐4.70)	<.01	2.03 (1.01–3.69)	.04
Hemodialysis	3.67 (2.11‐6.05)	<.01	2.48 (1.38‐4.27)	<.01
Peripheral vascular disease	3.01 (1.85‐4.90)	<.01	2.28 (1.35–3.73)	<.01
In‐stent restenosis	3.32 (1.66‐5.98)	<.01	3.17 (1.58–5.23)	<.01

Abbreviations: CI, confidence interval; CI‐TLR, clinically indicated target lesion revascularization; HR, hazard ratio.

## 4 DISCUSSION

The main findings of this study are as follows: (a) While the rate of incidence of late adverse events after X‐EES implantation was low, it continued to increase gradually over time up to 10 years after stent implantation; and (b) insulin‐treated diabetes, left ventricular dysfunction (LVEF <35%), hemodialysis, peripheral vascular disease, and in‐stent restenosis were independent risk factors of clinically indicated TLF.

Widespread use of second‐generation DES has resolved several clinical issues related to major adverse cardiac events.^12^, ^13^, ^14^ In our cohort, the TLF was dominated by clinical indicated TLR and the annual incidence of late clinical indicated TLR beyond 5 years after X‐EES implantation remained at 0.67% per year; this rate is much lower than that reported by previous studies.^6^, ^7^, ^12^, ^13^ Serruys et al, based on intravascular ultrasound (IVUS) findings, have suggested that X‐EES is more effective in reducing neointimal hyperplasia than the first‐generation DES.^15^ It is known that IVUS‐guided PCI is superior to angiography‐guided PCI with respect to reducing the risk of clinically indicated TLR or ST^16^; in this study, the IVUS‐guided PCI was performed for all the cases. Furthermore, as the risk factors of TLF in this cohort, insulin‐treated diabetes, hemodialysis, peripheral vascular disease, and in‐stent restenosis were those of clinically indicated TLR (Tables 4 and 5). However, achieving complete resolution in such patients is considered to be difficult, even with the IVUS‐guided PCI using second‐generation DES. Therefore, more careful follow‐up during routine clinical practice is required in such patients.

To date, several large‐scale registries of first‐generation DES have recorded annual VLST incidence rates of 0.21% to 0.53%.^4^, ^5^, ^16^ In contrast, some studies on X‐EES have reported annual VLST incidence rates of 0.13% to 0.18%, although this is based on data up to 5 years only.^6^, ^7^, ^13^ Thus, it is possible that the use of the X‐EES is associated with a lower incidence of long‐term events such as late ST and VLST compared with the first‐generation DES.^17^, ^18^ Multiple causes of ST have been generally reported to depend on the malapposition of the deployed stent, antiplatelet therapy, and clinical presentation (whether AMI or not).^19^, ^20^ As mentioned above, the use of IVUS‐guided PCI could have contributed to the lower TLR and ST rate^16^; thus, the low prevalence of ST in this study might be due to the use of IVUS. Specifically, in our hospital, the optimal stenting endpoint (minimal stent area > 5.5 mm^2^) is decided using IVUS imaging and is based on evidence from previous studies.^21^, ^22^ In three VLST cases of this study, the optimal stenting from the findings of the quantitative coronary angiography (QCA) and IVUS at the index PCI procedure was observed; however, in the other eight ST cases, malapposition and stent deformation were observed. All VLST cases had been prescribed with single antiplatelet therapy (aspirin 100 mg/day) at event occurrence, and two VLST cases had discontinued all antiplatelet therapy due to active bleeding. Of the patients who had acute and subacute ST in this study, all were AMI patients except one case who was prescribed with cilostazol due to clopidogrel intolerance. However, it is important to highlight here that the incidence rate of definite/probable ST was similar among AMI and non‐AMI cases. Also, in this study, the incidence of late or very late ST after the X‐EES implantation seemed to be low, but it gradually continued to increase up to 10 years. In the recent study, an individual's susceptibility to increasing ST rate is reported to be multifactorial and results from interactions between severe clinical factors, endothelial biology, hypersensitivity and/or inflammatory reactions, blood rheology, platelet reactivity, clotting factors, and physical and mechanical properties of the stent.^23^, ^24^ Therefore, the evidence‐based recommendation such as intravascular‐imaging‐informed percutaneous coronary intervention strategy combined with optimized antiplatelet therapy should be proposed for successful VLST clinical management.

## 5 LIMITATIONS

There are several important limitations to our study. First, this was a retrospective, single‐center study, which might have significantly affected some results, especially because confounding factors were not controlled for. Therefore, these results, which represent the post‐hoc analysis of a trial, should be used only for hypothesis generation. Next, no information on bleeding complications during the follow‐up period was available, and this outcome was, therefore, not analyzed.

## 6 CONCLUSIONS

In conclusion, the incidence rates of long‐term outcomes such as late TLR and definite/probable ST after X‐EES implantation were very low in this study; however, they gradually increased during the follow‐up period of 10 years. Larger trials based on clinical follow‐up are needed to confirm the use of X‐EES as an optimal long‐term strategy.

## CONFLICT OF INTEREST

The authors have no conflicts of interest to declare.

## AUTHOR CONTRIBUTIONS

Conceptualization: Hiroaki Matsuda

Data curation: Hiroaki Matsuda

Formal analysis: Hiroaki Matsuda

Investigation: Hiroaki Matsuda

Methodology: Hiroaki Matsuda

Project administration; Hiroaki Matsuda, Yoriyasu Suzuki

Writing – original draft preparation: Hiroaki Matsuda

Writing – review and editing: Hiroaki Matsuda, Yoriyasu Suzuki

All authors have read and approved the final version of the manuscript.

The corresponding author or manuscript guarantor will have to confirm that he/she had full access to all of the data in the study and takes complete responsibility for the integrity of the data and the accuracy of the data analysis.

## TRANSPARENCY STATEMENT

The lead author, Hiroaki Matsuda, affirms that this manuscript is an honest, accurate, and transparent account of the study being reported; that no important aspects of the study have been omitted; and that any discrepancies from the study as planned (and if relevant, registered) have been explained.

## Data Availability

The data that support the findings of this study are available from the corresponding author upon reasonable request.

## References

[1] Settler C, Wandel S, Allemann S, et al. Outcomes associated with drug‐eluting and bare‐metal stents: a collaborative network meta‐analysis. Lancet. 2007;370:937‐948. 17869634 10.1016/S0140-6736(07)61444-5

[2] Horst B, Rihal CS, Holmes DR Jr, et al. Comparison of drug‐eluting and bare‐metal stents for stable coronary artery disease. JACC Cardiovasc Interv. 2009;2:321‐328. 19463444 10.1016/j.jcin.2008.11.010

[3] Wiemer M, Serruys PW, Miquel‐Hebert K, et al. Five‐year long‐term clinical follow‐up of the XIENCE V everolimus eluting coronary stent system in the treatment of patients with de novo coronary artery lesions: the SPIRIT FIRST trial. Catheter Cardiovasc Interv. 2010;75:997‐1003. 20517959 10.1002/ccd.22428

[4] Costa JR, Sousa A, Moreina AC, et al. Incidence and predictors of very late (>or=4 years) major cardiac adverse events in the DESIRE (drug‐eluting stents in the real world)‐late registry. JACC Cardiovasc Interv. 2010;3:12‐18. 20129562 10.1016/j.jcin.2009.10.022

[5] Kimura T, Morimoto T, Nakagawa Y, et al. Very late stent thrombosis and late target lesion revascularization after sirolimus‐eluting stent implantation: five‐year outcome of the j‐Cypher Registry. Circulation. 2012;125:584‐591. 22203694 10.1161/CIRCULATIONAHA.111.046599

[6] Onuma Y, Miquel‐Hebert K, Serruys PW, SPIRIT II Investigators . Five‐year long‐term clinical follow‐up of the XIENCE V everolimus‐eluting coronary stent system in the treatment of patients with de novo coronary artery disease: the SPIRIT II trial. EuroIntervention. 2013;8:1047‐1051. 23339811 10.4244/EIJV8I9A161

[7] Gada H, Kirtane AJ, Newman W, et al. 5‐year results of a randomized comparison of XIENCE V everolimus‐eluting and TAXUS paclitaxel‐eluting stents: final results from the SPIRIT III trial (clinical evaluation of the XIENCE V everolimus eluting coronary stent system in the treatment of patients with de novo native coronary artery lesions). JACC Cardiovasc Interv. 2013;6:1263‐1266. 24239202 10.1016/j.jcin.2013.07.009

[8] Brener SJ, Kereiakes DJ, Simonton CA, et al. Everolimus‐eluting stents in patients undergoing percutaneous coronary intervention: final 3‐year results of the clinical evaluation of the XIENCE V Everolimus eluting coronary stent system in the treatment of subjects with de novo native coronary artery lesions trial. Am Heart J. 2013;166:1035‐1042. 24268218 10.1016/j.ahj.2013.08.030

[9] Tandjung K, Sen H, Lam MK, et al. Clinical outcome following stringent discontinuation of dual antiplatelet therapy after 12 months in real‐world patients treated with second‐generation zotarolimus‐eluting resolute and everolimus‐eluting Xience V stents: 2‐year follow‐up of the randomized TWENTE trial. J Am Coll Cardiol. 2013;61:2406‐2416. 23602769 10.1016/j.jacc.2013.04.005

[10] Kelly CR, Teirstein PS, Meredith IT, et al. Long‐term safety and efficacy of platinum chromium Everolimus‐eluting stents in coronary artery disease: 5‐year results from the PLATINUM trial. JACC Cardiovasc Interv. 2017;23:2392‐2400. 10.1016/j.jcin.2017.06.070 PMC5866729 29217001

[11] Cutlip DE, Windecker S, Mehran R, et al. Clinical end points in coronary stent trials: a case for standardized definitions. Circulation. 2007;115:2344‐2351. 17470709 10.1161/CIRCULATIONAHA.106.685313

[12] Smits PC, Vlachojannis GJ, McFadden EP, et al. Final 5‐year follow‐up of a randomized controlled trial of everolimus‐ and paclitaxel‐eluting stents for coronary revascularization in daily practice: the COMPARE trial (a trial of Everolimus‐eluting stents and paclitaxel stents for coronary revascularization in daily practice). JACC Cardiovasc Interv. 2015;8:1157‐1165. 26210806 10.1016/j.jcin.2015.03.028

[13] Shiomi H, Kozuma K, Morimoto T, et al. 7‐year outcomes of a randomized trial comparing the first‐generation sirolimus‐eluting stent versus the new‐generation everolimus‐eluting stent: the RESET trial. JACC Cardiovasc Interv. 2019;12:637‐647. 30947938 10.1016/j.jcin.2019.01.234

[14] Piccolo R, Bonaa KH, Efthimiou O, et al. Drug‐eluting or bare‐metal stents for percutaneous coronary intervention: a systematic review and individual patient data meta‐analysis of randomised clinical trials. Lancet. 2019;393:2503‐2510. 31056295 10.1016/S0140-6736(19)30474-X

[15] Serruys PW, Ruygrok P, Neuzner J, et al. A randomised comparison of an everolimus‐eluting coronary stent with a paclitaxel‐eluting coronary stent:the SPIRIT II trial. EuroIntervention. 2006;2:286‐294. 19755303

[16] Weisz G, Leon MB, Holmes DR Jr, et al. Five‐year follow‐up after sirolimus‐eluting stent implantation results of the SIRIUS (Sirolimus‐eluting stent in de‐novo native coronary lesions) trial. J Am Coll Cardiol. 2009;53:1488‐1497. 19389558 10.1016/j.jacc.2009.01.050

[17] Raber L, Magro M, Stefanini GG, et al. Very late coronary stent thrombosis of a newer‐generation everolimus‐eluting stent compared with early‐generation drug‐eluting stents: a prospective cohort study. Circulation. 2012;125:1110‐1121. 22302840 10.1161/CIRCULATIONAHA.111.058560

[18] Tada T, Byrne RA, Simunovic I, et al. Risk of stent thrombosis among bare‐metal stents, first‐generation drug‐eluting stents, and second‐generation drug‐eluting stents: results from a registry of 18,334 patients. JACC Cardiovasc Interv. 2013;6:1267‐1274. 24355117 10.1016/j.jcin.2013.06.015

[19] Brown J, O'Brien CC, Lopes AC, Kolandaivelu K, Edelman ER. Quantification of thrombus formation in malapposed coronary stents deployed in‐vitro through imaging analysis. J Biomech. 2018;71:296‐301. 29452756 10.1016/j.jbiomech.2018.01.044 PMC5878124

[20] Valgimigli M, Bueno H, Byrne RA, et al. 2017 ESC focused update on dual antiplatelet therapy in coronary artery disease developed in collaboration with EACTS: The Task Force for dual antiplatelet therapy in coronary artery disease of the European Society of Cardiology (ESC) and of the European Association for Cardio‐Thoracic Surgery (EACTS). Eur Heart J. 2018;39:213‐260. 28886622 10.1093/eurheartj/ehx419

[21] Song HG, Kang SJ, Ahn JM, et al. Intravascular ultrasound assessment of optimal stent area to prevent in‐stent restenosis after zotarolimus‐, everolimus‐, and sirolimus‐eluting stent implantation. Catheter Cardiovasc Interv. 2014;83:873‐878. 22815193 10.1002/ccd.24560

[22] Katagiri Y, De Maria GL, Kogame N, et al. Impact of post‐procedural minimal stent area on 2‐year clinical outcomes in the SYNTAX II trial. Catheter Cardiovasc Interv. 2019;93:E225‐E234. 30702187 10.1002/ccd.28105

[23] Waksman R, Kirtane AJ, Torguson R, et al. Correlates and outcomes of late and very late drug‐eluting stent thrombosis: results from DESERT (international drug‐eluting stent event registry of thrombosis). JACC Cardiovasc Interv. 2014;7:1093‐1102. 25240540 10.1016/j.jcin.2014.04.017

[24] Parodi G, La Manna A, Di Vito L, et al. Stent‐related defects in patients presenting with stent thrombosis: differences at optical coherence tomography between subacute and late/very late thrombosis in the mechanism of stent thrombosis (MOST) study. EuroIntervention. 2013;9:936‐944. 24384290 10.4244/EIJV9I8A157

